# Frequent loss of *FAM126A* expression in colorectal cancer results in selective *FAM126B* dependency

**DOI:** 10.1016/j.isci.2024.109646

**Published:** 2024-03-29

**Authors:** Shuang Li, Ting Han

**Affiliations:** 1 PTN Joint Graduate Program, School of Life Sciences, Peking University, Beijing 100871, China; 2 National Institute of Biological Sciences, Beijing 102206, China; 3 Tsinghua Institute of Multidisciplinary Biomedical Research, Tsinghua University, Beijing 102206, China

**Keywords:** Biological sciences, Molecular biology, Cancer systems biology

## Abstract

Most advanced colorectal cancer (CRC) patients cannot benefit from targeted therapy due to lack of actionable targets. By mining data from the DepMap, we identified *FAM126B* as a specific vulnerability in CRC cell lines exhibiting low *FAM126A* expression. Employing a combination of genetic perturbation and inducible protein degradation techniques, we demonstrate that FAM126A and FAM126B function in a redundant manner to facilitate the recruitment of PI4KIIIα to the plasma membrane for PI4P synthesis. Examination of data from TCGA and GTEx revealed that over 7% of CRC tumor samples exhibited loss of *FAM126A* expression, contrasting with uniform *FAM126A* expression in normal tissues. In both CRC cell lines and tumor samples, promoter hypermethylation correlated with the loss of *FAM126A* expression, which could be reversed by DNA methylation inhibitors. In conclusion, our study reveals that loss of *FAM126A* expression results in *FAM126B* dependency, thus proposing FAM126B as a therapeutic target for CRC treatment.

## Highlights

• Low FAM126A expression predicts FAM126B dependency in CRC cell lines and CDX
• FAM126 A/B redundancy underlies selective FAM126B dependency
• FAM126 A/B maintain plasma membrane PI4P essential for cell viability
• FAM126A promoter is frequently hypermethylated in CRC cell lines and tumors

## Graphical abstract

**Figure:**
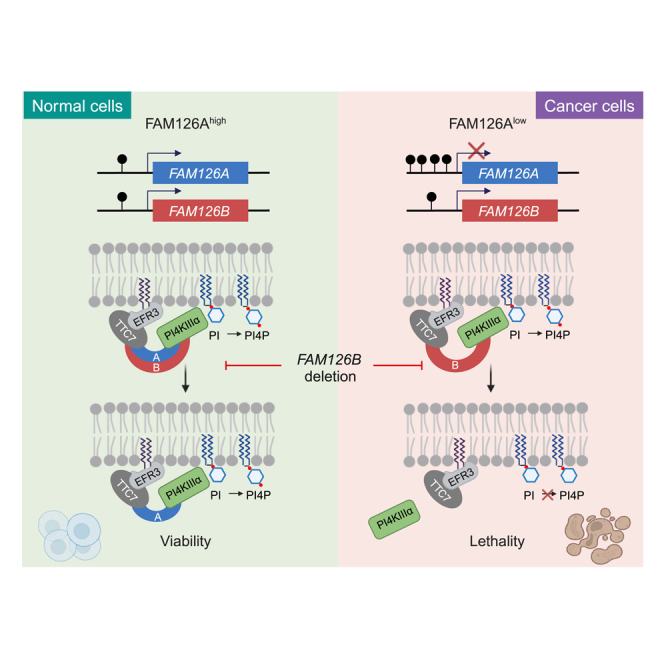

## Introduction

Colorectal cancer (CRC) is the third most common cancer worldwide, with an estimated 1.9 million new cases and 0.9 million deaths in 2020.^1^ Despite advances in early detection and treatment, CRC remains a significant public health challenge due to its high incidence and mortality rates, as well as the limited effectiveness of current treatments for advanced disease.^2^ Targeted therapy, which uses therapeutic agents to target oncogenic driver proteins that promote uncontrolled growth, division, or spreading of cancer cells, has become an important means of CRC treatment.^3^ For example, small molecules or monoclonal antibodies targeting epidermal growth factor receptor (EGFR), vascular endothelial growth factor (VEGF), and BRAF have a positive effect on improving the survival rate and quality of life for CRC patients.^4^^,^^5^ However, the long-term benefit of targeted therapy is hindered by acquired resistance leading to disease relapse.^6^^,^^7^ Moreover, only a fraction of oncogenic driver mutations are currently druggable; as a consequence, the majority of patients with advanced CRC cannot benefit from targeted therapy.^8^ Therefore, a better understanding of the molecular and genetic characteristics of CRC will enable the identification of new targets with the hope to broaden the scope of targeted therapy for CRC treatment. Synthetic lethality (SL) refers to a phenomenon that the perturbation of one of two genes can be tolerated, whereas the perturbation of both genes results in lethality.^9^ Originally described in model organisms, the concept of SL has been successfully applied to cancer treatment.^10^^,^^11^ For example, poly (ADP-ribose) polymerase (PARP) inhibitors cause cellular DNA damage, which can be repaired efficiently in normal cells. However, in cancer cells deficient in DNA repair due to *BRCA1/2* mutations, PARP inhibitors cause excessive DNA damage leading to cell death.^12^^,^^13^ PARP inhibitors have therefore been applied as a targeted therapy for cancers harboring *BRCA1/2* mutations.^14^ In addition, many new SL targets have been nominated and therapeutic agents targeting them are in or approaching clinical testing.^15^^,^^16^^,^^17^^,^^18^^,^^19^ The advances in cancer genomics and CRISPR (clustered regularly interspaced short palindromic repeats)-based gene perturbation methods have revolutionized the discovery of SL targets in cancer.^20^ For example, the Cancer Dependency Map (DepMap) project used CRISPR-Cas9 screening to uncover the fitness consequence of single-gene deletions (gene dependency) in hundreds of cancer cell lines.^21^ Coupled with multiple layers of genomic data, gene dependencies offer a valuable resource for identifying novel cancer targets and predictive biomarkers to enable precision medicine.^22^

Here we devised a bioinformatic method to identify SL interactions among gene paralogs in CRC cell lines and discovered that the expression level of *FAM126A* correlated with the essentiality of *FAM126B*. Using a combination of *in vitro* and *in vivo* approaches, we validated the SL interaction between *FAM126A* and *FAM126B* and demonstrated that loss of both FAM126A and FAM126B impaired plasma membrane phosphoinositide 4-phosphate synthesis to cause cell death. We further provide evidence that loss of *FAM126A* expression was prevalent in CRC tumors but not in normal tissues, suggesting that targeting FAM126B would be a safe and efficacy strategy to treat CRC with low *FAM126A* expression.

## Results

### Low *FAM126A* expression predicts *FAM126B* dependency in CRC cell lines

**Figure 1:**
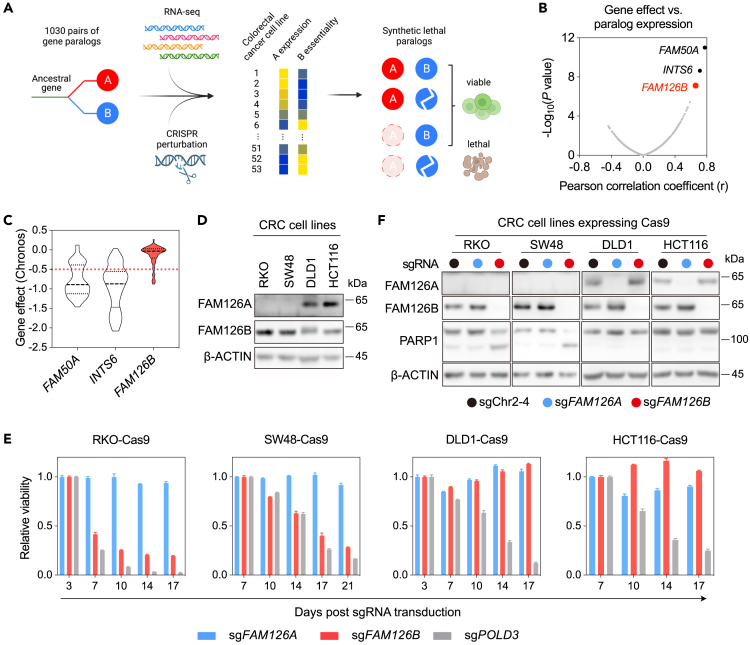
Discovery and validation of selective *FAM126B* dependency in CRC cell lines with low *FAM126A* expression (A) Strategy for discovering SL interactions between gene paralogs in CRC cell lines. (B) Scatterplot depicting the correlation between gene effects versus paralog expression levels among 1030 pairs of paralogs in 53 CRC cell lines. (C) Violin plot of gene effect (Chronos) for *FAM50A*, *INTS6*, and *FAM126B* among 53 CRC cell lines (DepMap Public 22Q1). (D) Detection of FAM126A and FAM126B proteins in indicated CRC cell lines. (E) Competitive cell growth assay after inactivation of *FAM126A*, *FAM126B*, or *POLD3* in indicated cell lines. All data were normalized to a control (sgChr2-4). *POLD3*, encoding DNA polymerase delta 3, is common essential gene. Data are the mean ± s.d. from three technical replicates. (F) Detection of PARP1 cleavage after FAM126A or FAM126B depletion in indicated cell lines.

Paralogs are different genes in the same species that arise from a common ancestral gene. They often inherit the essential function of their ancestor and can therefore display SL interactions.^23^ To reveal SL interactions among gene paralogs in CRC, we focused on 1,030 human gene families containing two paralogs with sequence identity greater than 50%.^24^ We obtained mRNA expression data and gene effect scores (Chronos)^25^ of these paralogs from the DepMap and analyzed the correlation of the Chronos scores of each gene with the expression levels of its paralog among 53 CRC cell lines (Figure 1A). By ranking the resulting Pearson correlation coefficients, three putative SL interactions with statistically significant correlations were identified: *FAM50A* dependency versus *FAM50B* expression, *INTS6* dependency versus *INTS6L* expression, and *FAM126B* dependency versus *FAM126A* expression (Figure 1B). The SL interaction between *FAM50A* and *FAM50B* has been described and experimentally validated in a previous study,^26^ thus benchmarking the effectiveness of our analysis.

To pursue SL interactions with potential therapeutic relevance, we examined the distribution of the Chronos scores of *FAM50A*, *INST6*, and *FAM126B* among 53 CRC cell lines in the DepMap. The average Chronos scores of *FAM50A* and *INTS6* were near −1, indicating that they were common essential genes. As targeting common essential genes often results in narrow therapeutic windows,^27^ we decided to focus on *FAM126B*, the Chronos scores of which followed a skewed distribution with a peak centered around 0, and a small tail extended toward −1 (Figure 1C).

We used four CRC cell lines—RKO, SW48, DLD1, and HCT116—to validate the finding that low *FAM126A* expression predicts *FAM126B* dependency. The levels of *FAM126A* mRNA in DLD1 and HTC116 were ∼100- and ∼800-fold higher, respectively, than the levels of *FAM126A* mRNA in RKO and SW48 (Figure S1A). Similarly, by western blotting, FAM126A protein was detectable in DLD1 and HCT116, but undetectable in RKO and SW48 (Figure 1D). In contrast, *FAM126B* was expressed at comparable levels among these four cell lines (Figures 1D and S1A). To examine the genetic dependencies of *FAM126A* and *FAM126B*, we identified sgRNAs that could efficiently deplete FAM126A and FAM126B (Figures 1F and S1B) and then used a competitive cell growth assay to measure the fitness effect following genetic deletion of *FAM126A* or *FAM126B*. CRC cells stably expressing Cas9 were infected with lentivirus co-expressing an sgRNA and a green fluorescent protein (GFP). Infected GFP-positive cells were mixed with cells without lentiviral infection and the percentages of GFP-positive cells were monitored by flow cytometer over time. An sgRNA targeting an intergenic region (sgChr2-4) was included as control for data normalization. As a positive control, transduction of an sgRNAs targeting *POLD3* (encoding a subunit of DNA polymerase δ) in all four cell lines caused fitness deficits (Figures 1E and S1C). *FAM126A* sgRNA transduction did not cause notable fitness deficits in all four CRC cell lines (Figures 1E and S1D). In contrast, RKO-Cas9 and SW48-Cas9 cells (FAM126A^low^) were depleted following *FAM126B* sgRNA transduction, whereas DLD1-Cas9 and HCT116-Cas9 cells (FAM126A^high^) were not depleted following *FAM126B* sgRNA transduction (Figures 1E and S1E). To exclude the possibility that the observed loss of cell fitness was due to an off-target effect of *FAM126B* sgRNA, we expressed an sgRNA-resistant *FAM126B* cDNA in RKO-Cas9 cells and observed that *FAM126B* sgRNA transduction no longer caused a reduction in cell fitness (Figures S1F and S1G).

To further explore the cellular outcomes of FAM126B depletion, we examined poly(ADP-ribose) polymerase-1 (PARP1) cleavage as a marker for apoptosis. FAM126B depletion induced PARP1 cleavage in FAM126A^low^ cell lines (RKO and SW48) but not in FAM126A^High^ cell lines (DLD1 and HCT116) (Figure 1F). Thus, depletion of FAM126B selectively triggered apoptosis in FAM126A^low^ cell lines.

**Figure 2:**
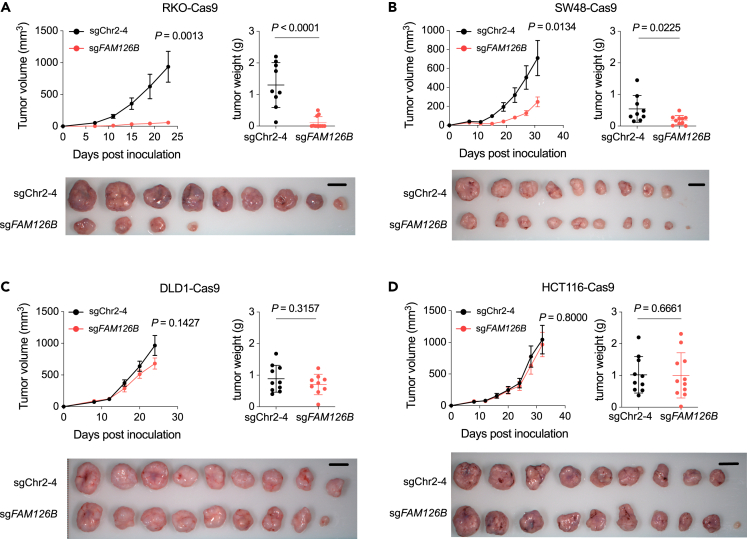
FAM126B depletion slows FAM126A^low^ tumor growth in nude mice (A–D) BALB/c NU mice were subcutaneously transplanted with indicated cell lines. Tumor volumes were measured at indicated time. Measurement of tumor weights and imaging of dissected tumors were performed at the end of the experiment. Data are the mean ± SEM. with *n* = 8–11 animals per group. Student’s t tests (two-tailed, unpaired) were used to determine the statistical significance of the differences in tumor volume and tumor weight.

We further extended our analysis of *FAM126B* dependency from *in vitro* to *in vivo* by subcutaneously inoculating control or FAM126B-depleted CRC cells into nude mice. FAM126B depletion significantly inhibited the growth of tumors derived from FAM126A^low^ CRC cell lines RKO and SW48 (Figures 2A and 2B). In contrast, tumors derived from FAM126A^high^ CRC cell lines DLD1 and HCT116 (Figures 2C and 2D) were not affected by FAM126B depletion. Taken together, we conclude that *FAM126B* is a selective vulnerability of CRC cell lines with low FAM126A expression both *in vitro* and *in vivo*.

### *FAM126* paralog redundancy underlies selective *FAM126B* dependency

**Figure 3:**
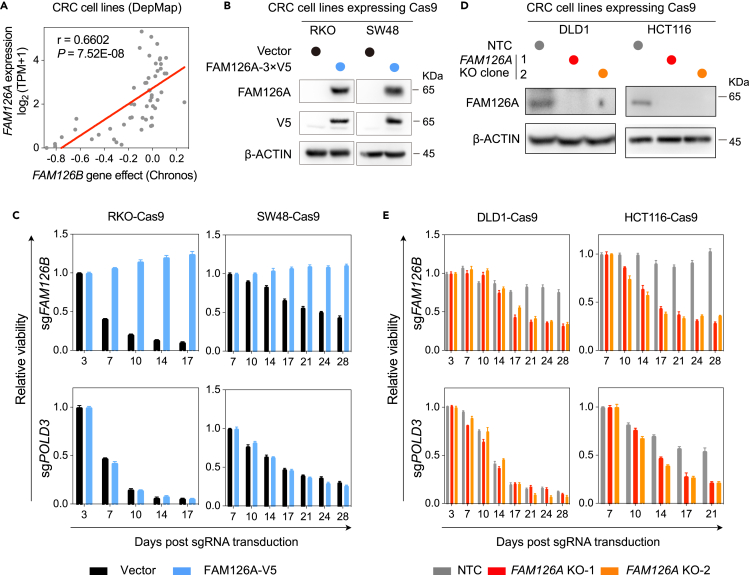
Loss of *FAM126A* expression causes *FAM126B* dependency in CRC cell lines (A) Scatterplot depicting the correlation between *FAM126A* expression and *FAM126B* gene effect. TPM stands for transcripts per million clean reads. Pearson correlation coefficient (r) and *p* value were indicated on the plot. Linear regression was represented by the red line. (B) Detection of FAM126A and FAM126A-V5 in indicated cell lines by western blotting. (C) Competitive cell growth assay after inactivation of *FAM126B* or *POLD3* in RKO-Cas9 and SW48-Cas9 cells expressing vector or FAM126A-V5. Data are the mean ± SD. from three technical replicates and normalized to control (sgChr2-4). (D) Verification of *FAM126A* knock out clones from DLD1 and HCT116. (E) Competitive cell growth assay after inactivation of *FAM126B* or *POLD3* in *FAM126A* knock out clones relative to control cells expressing non-targeting control (NTC) sgRNA. Data are the mean ± SD from three technical replicates and normalized to control (sgChr2-4).

The significant correlation between *FAM126A* expression and *FAM126B* dependency (Figures 3A and S2A) among 53 CRC cell lines suggests that FAM126A and FAM126B are functionally redundant and that low expression of *FAM126A* may be a cause of *FAM126B* dependency. To test this hypothesis, we expressed FAM126A with a 3×V5 tag at its C terminus in FAM126A^low^ cell lines (RKO and SW48) (Figure 3B). Using the competitive cell growth assay, we observed that restoration of FAM126A expression in FAM126A^low^ cell lines resulted in the bypass of *FAM126B* dependency (Figure 3C). PARP1 cleavage in FAM126A^low^ cell lines following FAM126B depletion was also abrogated by FAM126A-3×V5 expression (Figure S2B). Moreover, we isolated multiple independent *FAM126A* knockout clones from FAM126A^high^ cell lines (Figure 3D) and observed these clones became dependent on *FAM126B* (Figures 3E and S2C).

To unbiasedly explore alterations of genetic dependencies following *FAM126A* perturbation, we performed two parallel genome-wide CRISPR-Cas9 screens: (1) RKO versus RKO overexpressing FAM126A-3×V5, (2) DLD1 parental versus *FAM126A* knockout cells. After lentiviral transduction of the sgRNA library, we propagated cells for 3 weeks and then performed next-generation sequencing to quantify the abundance of each sgRNA in surviving cells. By the MAGeCK (model-based analysis of genome-wide CRISPR/Cas9 knockout) algorithm, we found that *FAM126B* was a top-depleted gene both in RKO cells relative to RKO cells overexpressing FAM126A-3×V5, and in DLD1 *FAM126A* knockout cells relative to parental cells (Figures S2D–S2E). By comparing the top ten depleted genes in the above two screens, the only intersection was *FAM126B* (Figure S2F). Taken together, our results demonstrate that loss of *FAM126A* expression is the cause of *FAM126B* dependency among CRC cell lines.

### FAM126B degradation depletes plasma membrane PI4P in FAM126A^low^ CRC cells

FAM126A is known to localize phosphatidylinositol 4-kinase IIIα (PI4KIIIα) to the inner leaflet of plasma membrane (PM). Proper localization is necessary for PI4KIIIα to catalyze the synthesis of phosphatidylinositol 4-phosphate (PI4P).^28^ PI4P is the key anionic lipid that specifies PM identity and supports some of its key functions by recruiting effector proteins.^29^ Moreover, PI4P is the precursor to key signaling lipids phosphatidylinositol 4,5-bisphosphate and phosphatidylinositol (3,4,5)-trisphosphate.^30^ We therefore examined whether depletion of FAM126B in FAM126A^low^ CRC cells could affect PM PI4KIIIα localization and PI4P levels.

**Figure 4:**
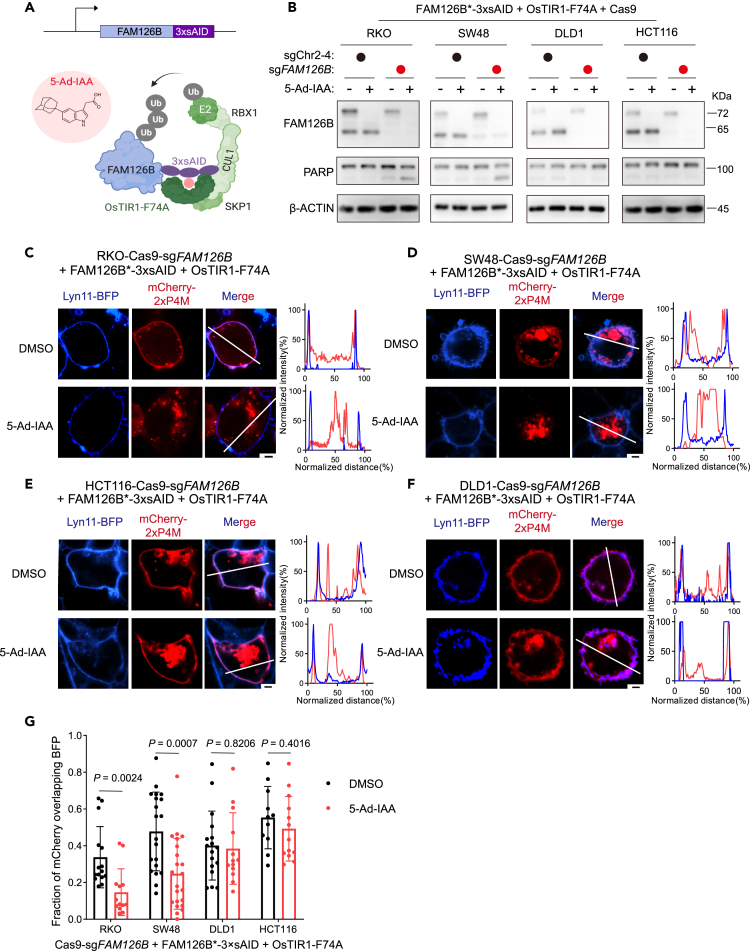
Induced FAM126B degradation depletes plasma membrane PI4P pool (A) Schematic illustration of induced FAM126B degradation using auxin-inducible degron (AID) system. (B) Detection of FAM126B degradation and PARP1 cleavage in indicated cell following treatment with DMSO or 250 ng/mL 5-Ad-IAA for 24 h. (C–F) Detection of cellular PI4P by transiently transfecting mCherry-2×P4M probe into indicated cells. Lyn11-BFP is a plasma membrane marker. Scale bar: 2.5 μm. Correlation between mCherry and BFP signals along indicated lines were plotted. (G) Quantification of the correlation between mCherry and BFP signals in indicated cells following DMSO or 5-Ad-IAA treatment. Each dot represents one cell. *p* values were computed by Student’s t test (two tailed, unpaired).

In order to deplete FAM126B in a rapid and synchronized manner, we adopted an improved auxin-induced degron (AID) system.^31^ We first expressed FAM126B-3×AID at the near-endogenous level together with an F box protein OsTIR1 that harbors a mutation (F74A) at its auxin-binding pocket. OsTIR1-F74A forms an E3 ubiquitin ligase complex (SCF^TIR1-F74A^), which binds to a bulky analog of auxin—5-adamantyl-indole-3-acetic acid (5-Ad-IAA)—to induce the degradation FAM126B-3×AID via the ubiquitin-proteasome system (Figure 4A). We then knocked out endogenous *FAM126B* so that the only FAM126B in the resulting cells were FAM126B-3×AID. Following cell line engineering as described previously, 5-Ad-IAA treatment induced rapid depletion of FAM126B-3×AID in both FAM126A^high^ and FAM126A^low^ cell lines. However, PARP1 cleavage and loss of cell viability were only observed in FAM126A^low^ cell lines RKO and SW48 but not in FAM126A^high^ cell lines DLD1 and HCT116 (Figures 4B, S3A, and S3B).

To determine whether FAM126B degradation in FAM126A^low^ cells affected PI4KIIIα PM localization, we separated cell lysates into crude fractions containing membrane or cytosol (Figure S3C). We found that degradation of FAM126B in RKO cells resulted in reduced levels of PI4KIIIα in the membrane fraction (Figure S3D). To visualize cellular PI4P following FAM126B degradation, we used mCherry-2×P4M as a PI4P probe. P4M is a specific PI4P binding domain of the SidM protein from *Legionella pneumophila*. Fusing two P4M domains in tandem was shown to enhance binding to PI4P.^32^^,^^33^ Expression of mCherry-2×P4M labeled both PM (colocalizing with membrane-targeted Lyn11-BFP) and the Golgi apparatus (Figures 4C–4F). To determine the specificity of the mCherry-2×P4M probe, we expressed a membrane-targeted PI4P phosphatase (Lyn11-Sac1) and found that PM mCherry signals were lost, whereas Golgi mCherry signals were not affected (Figures S3E and S3F). Thus, PM localization of mCherry-2xP4M was dependent on PI4P. We next used the mCherry-2×P4M probe to visualize PI4P in CRC cells. Degradation of FAM126B significantly depleted the PM pool of PI4P in FAM126A^low^ cell lines RKO and SW48, but not in FAM126A^high^ cell lines DLD1 and HCT116 (Figures 4C–4G, S4A, S4B, S5A, and S5B). In conclusion, degradation of FAM126A in CRC cell lines with low FAM126A expression impaired PM PI4KIIIα localization and subsequently depleted the PM PI4P pool.

### PI4KIIIα PM tethering bypasses *FAM126B* dependency in FAM126A^low^ CRC cells

**Figure 5:**
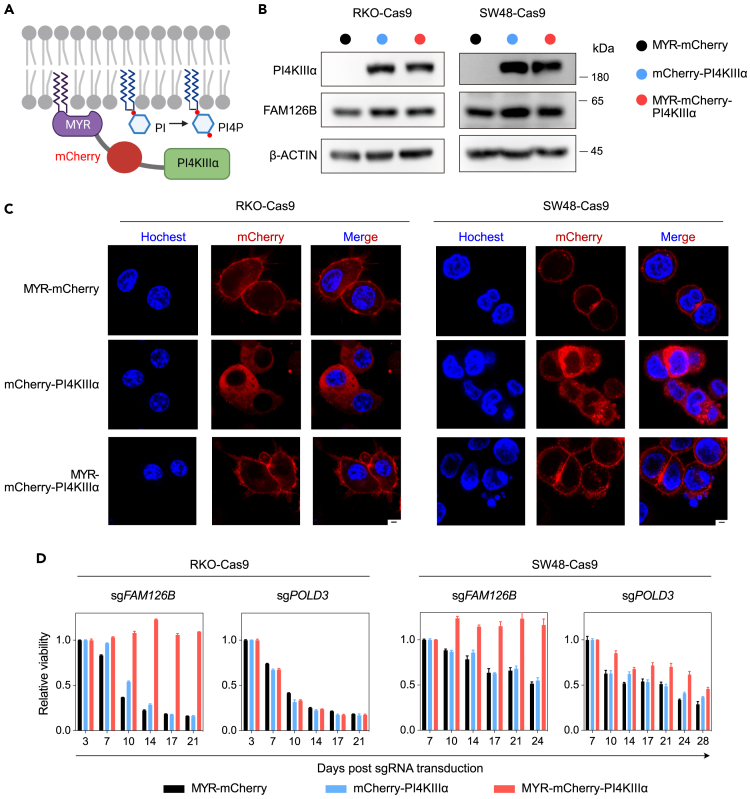
Tethering PI4KIIIα to the plasma membrane rescues cell viability following FAM126B depletion in FAM126A^low^ CRC cells (A) Strategy for tethering PI4KIIIα to the plasma membrane via the addition of a myristoylation signal (MYR). (B) Detection of PI4KIIIα and FAM126B in indicated cells expressing MYR-mCherry, mCherry-PI4KIIIα, or MYR-mCherry-PI4KIIIα. (C) Subcellular localization of MYR-mCherry, mCherry-PI4KIIIα, or MYR-mCherry-PI4KIIIα in indicated cell lines visualized by confocal imaging. Hochest staining was used to visualize nuclei. Scale bar: 2.5 μm. (D) Competitive cell growth assay after inactivation of *FAM126B* or *POLD3* in indicated cell lines expressing MYR-mCherry, mCherry-PI4KIIIα, or MYR-mCherry-PI4KIIIα. Data are the mean ± SD from three technical replicates and normalized to control (sgChr2-4).

The PI4KIIIα protein is encoded by the gene *PI4KA*. In CRC cell lines with either high or low expression levels of FAM126A, depletion of *PI4KA* resulted in reduced cell viability (Figures S6A and S6B). Moreover, the average Chronos score of *PI4KA* among 53 CRC cell lines in the DepMap was around −1 (Figure S4C), indicating *PI4KA* as a common essential gene. These observations prompted us to test whether reduced plasma membrane PI4KIIIα localization was the cause of cell death in FAM126A^low^ CRC cells following FAM126B depletion. We fused PI4KIIIα with an N-terminal myristoylation motif and mCherry (MYR-mCherry-PI4KIIIα) to artificially tether PI4KIIIα to PM. As controls, we generated constructs expressing MYR-mCherry or mCherry-PI4KIIIα (Figure 5A). These constructs were introduced into RKO-Cas9 and SW48-Cas9. Western blotting indicated that MYR-mCherry-PI4KIIIα and mCherry-PI4KIIIα were expressed at comparable levels (Figure 5B). In both cell lines, MYR-mCherry and MYR-mCherry-PI4KIIIα predominately localized to PM, whereas mCherry-PI4KIIIα predominately localized to the cytoplasm (Figure 5C). Next, we used competitive cell growth assay to examine whether MYR-mCherry-PI4KIIIα could rescue cell death following FAM126B depletion. Whereas RKO-Cas9 and SW48-Cas9 cells expressing MYR-mCherry or mCherry-PI4KIIIα were still sensitive to the transduction of *FAM126B* sgRNA, MYR-mCherry-PI4KIIIα expression rendered these cell lines resistant (Figure 5D). The rescuing effect of MYR-mCherry-PI4KIIIα was specific to *FAM126B* sgRNA, because loss of cell viability following *POLD3* sgRNA transduction was not rescued (Figure 5D). Taken together, these results indicate that failure to localize PI4KIIIα to PM is the cause of *FAM126B* dependency in FAM126A^low^ CRC cells.

### Loss of FAM126A expression is associated with promoter hypermethylation in CRC

**Figure 6:**
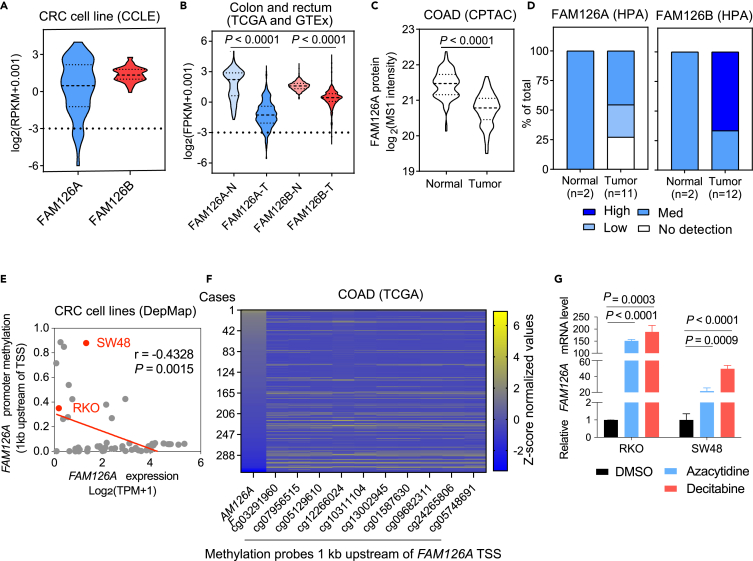
Promoter hypermethylation silences *FAM126A* expression in a subset of CRC cell lines and primary tumors (A) Violin plot depicting the distribution of *FAM126A* and *FAM16B* expression in 57 CRC cell lines from CCLE. (B) Violin plot depicting distribution of *FAM126A* and *FAM126B* expression in CRC tumor samples (*n* = 637) versus normal tissue samples (*n* = 356). Data were obtained from TCGA and GTEx and filtered by log_2_(FPKM+0.001)> -9. (C) Violin plot depicting the distribution of FAM126A protein expression in CRC tumor samples (*n* = 97) versus normal tissue samples (*n* = 100). Data were obtained from CPTAC. (D) Distribution of FAM126A and FAM126B IHC staining intensities in CRC tumor samples and normal tissue samples. Data were obtained from HPA. (E) Scatterplot depicting the correlation between *FAM126A* expression and promoter methylation in CRC cell lines from DepMap. Pearson correlation coefficient (r) and *p* value were indicated. (F) Heatmap depicting *FAM126A* expression levels and *FAM126A* promoter methylation levels. DNA methylation data were obtained from TCGA Methylation 450k and promoter region were determined according to Mexpress. After excluding NA data, 321 cases were used for analysis. (G) Effect of azacytidine and decitabine on *FAM126A* expression. RKO or SW48 cells were treated with 4 μM azacytidine or 20 μM decitabine for 72 h before qPCR analysis of *FAM126A* expression. Student’s t tests (two-tailed, unpaired) were used to determine the statistical significance. Data were the mean ± SD of three biological replicates.

To explore the relevance of our findings, we examined the prevalence and potential cause of low FAM126A expression in CRC cell lines and primary tumors. By analyzing mRNA expression data of the Cancer Cell Line Encyclopedia (CCLE), we found that the expression levels of FAM126B were distributed within a narrow range, whereas the expression levels of FAM126A were distributed over a much wider range in CRC cell lines (Figure 6A). Using a cutoff of log2(FPKM+0.001) <-3, 10.5% of CRC cell lines could be defined as FAM126Alow. To verify the prevalence of low *FAM126A* expression in CRC cell lines, we measured the levels of FAM126A protein in nine CRC cell lines and two normal cell lines (293T and HaCaT) by western blotting (Figures S7A and S7B). In addition to RKO and SW48, LS513 and HT29 did not express detectable levels of FAM126A. In contrast, FAM126A was readily detectable in SW480, LoVo, 293T, and HaCaT. Intermediate levels of FAM126A were detected in CACO2 and HT15. As a further validation of our findings, we depleted FAM126B in LS513 (FAM126A^low^) and LoVo (FAM126A^high^) and observed that FAM126B depletion reduced the viability of LS513 but exhibited a much smaller effect on the viability of LoVo (Figures S7C and S7D).

By analyzing mRNA expression data from Genotype-Tissue Expression database (GTEx) and The Cancer Genome Atlas (TCGA), we observed that the expression levels of *FAM126A* were significantly lower in CRC tumors relative to normal tissues (Figure 6B). Using a cutoff of log2(FPKM+0.001) <-3, 7.4% of CRC tumor samples could be defined as FAM126Alow, whereas none of the normal samples passed the cutoff. Although the expression levels of *FAM126B* were also lower in CRC tumors than in normal tissues, the differences were not as large as the differences in *FAM126A*. By analyzing proteomic data from CPTAC (The National Cancer Institute’s Clinical Proteomic Tumor Analysis Consortium), we found that the protein level of FAM126A was significantly lower in CRC tumors than in normal colon tissues (Figure 6C). We further investigated the immunohistochemistry data from HPA (Human Protein Atlas). Consistent with the CPTAC, FAM126A but not FAM126B protein levels were lower in CRC tumors than in normal colon tissues (Figure 6D).

Finally, we investigated the potential mechanism responsible for low *FAM126A* expression in CRC. In both CRC cell lines from CCLE and primary CRC tumor samples from TCGA, *FAM126A* expression was negatively correlated with the DNA methylation levels of its promoter region (Figures 6E and 6F). However, there were also cell lines with low *FAM126A* expression but low promoter DNA methylation, suggesting the existence of other epigenetic mechanisms responsible for the silencing of *FAM126A* expression. In order to test whether *FAM126A* promoter hypermethylation could be a cause of low *FAM126A* expression, we treated RKO and SW48 cells (FAM126A^low^) with DNA methylation inhibitors azacytidine and decitabine, which induced degradation of DNA methyltransferase DNMT1 as previously described (Figure S7E).^34^^,^^35^ By qPCR, we observed that azacytidine or decitabine treatment activated *FAM126A* expression but not *FAM126B* expression (Figures 6C and S7F). Taken together, promoter DNA methylation could be a cause of *FAM126A* silencing in CRC.

Microsatellite instability (MSI) is a key biomarker for colorectal cancer (CRC), accounting for approximately 15% of all CRC cases.^36^ Considering the importance of MSI, we analyzed whether there was an association between *FAM126A* expression and MSI. By analyzing data from CCLE and TCGA, we found that there was no difference in *FAM126A* or *FAM126B* expression in MSI versus MSS (microsatellite-stable) colorectal cancers (Figures S8A and S8C). The Chronos scores of *FAM126A* and *FAM126B* were not significantly different between MSI and MSS CRC cell lines (Figure S8D). Moreover, *FAM126A* expression and *FAM126B* Chronos scores were significantly correlated in both MSI and MSS CRC cell lines (Figures S8D and S8E).

## Discussion

SL interactions have been a topic of great interest in cancer research with the promise of identifying new molecular targets for precision anti-cancer therapy.^9^^,^^10^ Although SL interactions with commonly mutated tumor suppressor genes such as *P53*, *Rb*, and *PTEN* have remained elusive, the combination of high-throughput experimental determination of gene essentiality and newly developed computational algorithms have revealed a large collection of SL candidates,^17^^,^^19^^,^^37^^,^^38^^,^^39^^,^^40^^,^^41^^,^^42^^,^^43^^,^^44^ some of which are being or approaching being tested in clinical trials. For example, *MTAP* (encoding methylthioadenosine phosphorylase) is located in proximity to the tumor suppressor gene *CDKN2A* in the genome and thus often co-deleted with *CDKN2A* in cancer cells. Loss of *MTAP* results in the accumulation of 5′-methylthioadenosine, which compromises the activity of protein arginine methyltransferase 5 (PRMT5). Thus *MTAP*-deleted cancer cells are more sensitive to PRMT5 inhibitors.^15^^,^^16^ More recently, CRISPR screening in large panels of cancer cell lines revealed WRN—encoding Werner syndrome helicase—as a selective essential gene in microsatellite unstable cancers.^17^^,^^45^ TA-dinucleotide repeats are highly unstable and undergo large-scale expansions in microsatellite unstable cancers, resulting in the formation of DNA secondary structures resolved by WRN. In the absence of WRN, expanded TA-dinucleotide repeats are unresolved, leading to excessive DNA damage.^42^ From these two examples, studies of SL interactions have not only provided candidate targets for cancer therapeutics but also revealed hidden interactions between biological pathways.

SL interactions are more frequently observed between gene paralogs.^46^ Paralogs are duplicated from a common ancestral gene and evolve unique functions.^47^ However, paralogs often inherit the functions of their ancestral gene, likely as a mechanism to buffer against deleterious mutations in genes whose products mediate essential functions.^48^ The first reported paralog SL interaction in cancer involves *ENO1* and *ENO2*, encoding the glycolytic enzyme enolase. *ENO1* is a recurrently deleted passenger gene in glioblastoma. Loss of *ENO1* sensitizes glioblastoma cells to ENO2 inhibition.^49^ Since this seminal study, additional SL interactions involving paralogs that are recurrently mutated, deleted, or silenced in cancer have been reported.^50^ In this study, we discovered the SL interaction between *FAM126A* and *FAM126B*. FAM126A and FAM126B share a common function by recruiting PI4KIIIα to PM to catalyze the synthesis of PI4P. Cells tolerate the loss of either FAM126A or FAM126B. However, when both are lost, PM PI4P pool is depleted, resulting in cell death. The localization of PI4KIIIα to PM also requires two additional family of proteins, TTC7 and EFR3, both of which are encoded by two paralogs, *TTC7A/B* and *EFR3A/B*.^28^^,^^51^ Similar to *FAM126A* and *FAM126B*, low expression of *TTC7B* and *EFR3B* are prevalent among cancer cell lines, resulting in selective genetic dependency of their paralogs, *TTC7A* and *EFR3A*, respectively^26^ (Figures S9A–S9D). These observations suggest that PI4KIIIα localization is a heavily guarded process against genetic perturbations.

Whereas low *FAM126A* expression is prevalently observed in CRC, *FAM126B* is more uniformly expressed, suggesting these two genes have evolved unique functions. For *in vitro* cancer cell proliferation or *in vivo* tumor growth in immunodeficient mice, FAM126A and FAM126B do not display different functions, suggesting that such unique function does not involve autonomous cell growth or survival. Future studies in the context of tumor-host interaction and in the setting of therapeutic intervention may provide clues to the answer of this question.

Discovery of selective *FAM126B* dependency in FAM126A^low^ CRC provides an opportunity for developing new targeted therapy for CRC. Although our genetic perturbation of *FAM126B* in FAM126A^low^ CRC cell lines and cell line-derived xenograft models demonstrated antitumor activity of FAM126B targeting, two issues need to be resolved in order to translate our findings into clinical testing. First, to ensure the safety of FAM126B targeting, we need to test the effect of FAM126B targeting in a variety of primary cells derived from human beings. Second, a therapeutic agent needs to be developed to specifically target FAM126B. Although the N-terminal folded domains of FAM126A and FAM126B are highly similar, their C-terminal disordered regions are highly divergent. New technologies such as molecular glue degraders may provide a path to target the disordered region of FAM126B.^52^

### Limitations of the study

For functional studies, our study uses human cancer cell lines and cell-line-derived-xenograft models, which may not fully mimic human tumors. Although our study reveals that the loss of plasma membrane PI4P is the underlying cause of cell death following FAM126 perturbation, it remains unclear how the reduction of plasma membrane PI4P leads to cell death.

## STAR★Methods

### Key resources table

**Table:** 

REAGENT or RESOURCE	SOURCE	IDENTIFIER
**Antibodies**		

Anti-FAM126B	Novus Biologicals	Cat#: NBP1-81636 RRID: AB_11031139
Anti-FAM126A	Proteintech	Cat#: 26243-1-AP RRID: AB_2880443
Anti-FAM126A	Sino Biological	Cat#: 206234-T34 RRID: AB_2938777
Anti-PARP	Cell Signaling Technology	Cat#: 9542S RRID: AB_2160739
Anti-V5-HRP	Sigma	Cat#: V2260 RRID: AB_261857
Anti-PI4KIIIα	Cell Signaling Technology	Cat#: 4902S RRID: AB_2164029
Anti-DNMT1	Sino Biological	Cat#: 201485-T42 RRID: AB_2938778
Anti-β-ACTIN-HRP	Huaxingbio	Cat#: HX18271 RRID: AB_2938779
Anti-Rabbit IgG-HRP	Cell Signaling Technology	Cat#: 7074S RRID: AB_2099233

**Chemicals, peptides, and recombinant proteins**		

5-azacytidine	MedChemExpress	HY-10586
Decitabine	MedChemExpress	HY-A0004
5-Ad-IAA	Tokyo Chemical Industry	A3390
Polybrene	Yeasen	40804ES76
PEI	Yeasen	40816ES02
Puromycin	InvivoGen	ant-pr-1
Blasticidin	InvivoGen	ant-b1-1
Hygromycin B	Sigma	V900372-1G

**Critical commercial assays**		

Bicinchoninic acid (BCA) kit	Beyotime Biotechnology	P0009
CellTiter-Glo® (CTG)	Promega	G7571

**Deposited data**		

NGS results from CRISPR screen	This study	https://ngdc.cncb.ac.cn/bioproject/browse/PRJCA024139

**Experimental models: Cell lines**		

293T	Dr. Deepak Nijhawan’s lab at University of Texas Southwestern Medical Center	N/A
RKO	Dr. Deepak Nijhawan’s lab at University of Texas Southwestern Medical Center	N/A
SW48	Dr. Deepak Nijhawan’s lab at University of Texas Southwestern Medical Center	N/A
DLD1	Dr. Deepak Nijhawan’s lab at University of Texas Southwestern Medical Center	N/A
HCT116	Dr. Deepak Nijhawan’s lab at University of Texas Southwestern Medical Center	N/A
LoVo	Dr. Deepak Nijhawan’s lab at University of Texas Southwestern Medical Center	N/A
HT29	Dr. Xiaodong Wang’s lab at NIBS, Beijing	N/A
LS513	MeisenCTCC	CTCC-ZHYC-0227
CACO2	Cell Resource Center, Peking Union Medical College	1101HUM-PUMC000100
HCT15	Cell Resource Center, Peking Union Medical College	1101HUM-PUMC000247
SW480	Cell Resource Center, Peking Union Medical College	1101HUM-PUMC000166

**Experimental models: Organisms/strains**		

BALB/c-Nu	GemPharmatech	D000521

**Oligonucleotides**		

sgRNA Targeting sequences for Chr2-2: GGTGTGCGTATGAAGCAGTG	This paper	N/A
sgRNA Targeting sequences for Chr2-4: GCAGTGCTAACCTTGCATTG	This paper	N/A
sgRNA Targeting sequences for *FAM126B*: ACCATTCTTCCACAACACAA	This paper	N/A
sgRNA Targeting sequences for *FAM126B*-2: ACCATTCTTCCACAACACAA	This paper	N/A
sgRNA Targeting sequences for *FAM126A*: ATCTCTCTATAAAGTTATCC	This paper	N/A
sgRNA Targeting sequences for *FAM126A-2*: GAAAGTACTTACCTCACTTTG	This paper	N/A
sgRNA Targeting sequences for NTC: GAACTCGTTAGGCCGTGAAG	This paper	N/A
sgRNA Targeting sequences for *POLD3*: GGTTCCGTGACAGACACTGT	This paper	N/A
sgRNA Targeting sequences for *POLD3*-2: GGTTCCGTGACAGACACTGT	This paper	N/A
sgRNA Targeting sequences for *PI4KA*: GATAGTCTGTTATTACCTGT	This paper	N/A
sgRNA Targeting sequences for *PI4KA*-2: GCTGGCCAGAAGAATGGTACG	This paper	N/A
Forward qPCR primer sequences for *ACTB:* TCCCCTCCTTATCCAAGCCT	This paper	N/A
Reverse qPCR primer sequences for *ACTB*: ATGCTGACACAATGCCCCTT	This paper	N/A
Forward qPCR primer sequences for *FAM126A*: CACGAGTCGAGGTCCTGC	This paper	N/A
Reverse qPCR primer sequences for *FAM126A*: TCCTCCACAACCCCTTTCTC	This paper	N/A
Forward qPCR primer sequences for *FAM126B*: CATGTACGTTGCTATCCAGGC	This paper	N/A
Reverse qPCR primer sequences for *FAM126B*: CTCCTTAATGTCACGCACGAT	This paper	N/A

**Software and algorithms**		

R	Bell Laboratories	https://www.r-project.org/
R Studio	Ursa Labs	https://www.rstudio.com/categories/rstudio-ide/
MAGeCK	NIH	https://hpc.nih.gov/apps/MAGeCK.html
FlowJo	FlowJo	https://www.flowjo.com/
ImageJ	NIH	https://imagej.net/NIH_Image
Just Another Colocalization Plugin (JACOP)	ImageJ	https://imagej.nih.gov/ij/
FlowJo	BD	https://www.flowjo.com/
GraphPad Prism 8	Graphpad	https://www.graphpad.com/scientific-software/prism/

### Resource availability

#### Lead contact

Further information and requests for data and code should be directed to and will be fulfilled by the lead contact, Ting Han (hanting@nibs.ac.cn).

#### Materials availability

This study did not generate new animal lines or unique reagents.

#### Data and code availability

• Data: All sequencing data that support the findings of this study is publicly available (https://ngdc.cncb.ac.cn/bioproject/browse/PRJCA024139).
• Code: Not applicable.
• Any additional information required to reanalyze the data reported in this work paper is available from the lead contact upon request.

### Experimental model and study participant details

#### Animals

The source of female BALB/c-Nu mice (8–10-week-old) is provided in key resources table. All experiments were performed following the national guidelines for housing and care of laboratory animals (Ministry of Health, China) and the protocol is in compliance with institutional regulations after review and approval by the Institutional Animal Care and Use Committee at NIBS, Beijing. All mice were provided with food and water *ad libitum*, and housed under humidity (50% ± 10% relative humidity) and temperature (23 ± 1°C) controlled conditions on a 12-h light/dark cycle (light between 09:00 and 21:00). For *in vivo* tumor challenge experiments, 4×10^6^ CRC cells in 125 μL Dulbecco’s phosphate-buffered saline (DPBS, Gibco) were inoculated to 8–10-week-old female BALB/c-Nu mice. Tumor length (L) and width (W) were determined by Vernier caliper at the indicated times, and tumor volumes were calculated by L×W^2^×0.5.

#### Cell lines

Sources of cell lines used in this study are provided in key resources table. All cell lines were cultured at 37°C in humified incubators with 5% CO_2_. All culture media were supplemented with 10% fetal bovine serum (FBS, Gibco), 2 mM L-glutamine (Invitrogen), and 1% penicillin-streptomycin solution (Gibco). RKO, SW48, DLD1, HCT116, LS513, HT29, SW480, HCT15, CACO2 cell lines were cultured using the RPMI-1640 medium (Gibco). 293T, HaCaT cell lines were cultured using the DMEM medium (Gibco). Routine PCR test was used to ensure these cell lines were free of mycoplasma contamination.

### Method details

#### Antibodies and western blotting

The following antibodies were used by dilution in 5% (w/v) skim milk in PBST (PBS with 0.1% Tween 20): anti-FAM126B (Novus Biologicals, NBP1-81636, 1:1,000), anti-β-Actin-HRP (Huaxingbio, HX18271, 1:10,000), anti-V5-HRP (Sigma, V2260, 1:10,000), anti-FAM126A (Proteintech, 26243-1-AP, 1:500), anti-FAM126A (Sino Biological,206234-T34,1:1,000), anti-PARP1 (Cell Signaling Technology, 9542S, 1:1,000), anti-PI4KⅢα (Cell Signaling Technology, 4902S, 1:500), anti-DNMT1 (Sino Biological, 201485-T42, 1:1,000), anti-ATPA1 (Abclonal, A11683, 1:1,000), anti-GAPDH-HRP (Abcam, ab204481, 1:1,000), and anti-Rabbit IgG-HRP (Cell Signaling Technology, 7074S, 1:5,000). Total protein was extracted with SDS lysis buffer (20 mM HEPES-NaOH, pH 8.0, 10 mM NaCl, 2 mM MgCl_2_, and 1% SDS) freshly supplemented with 0.5 units/mL Benzonase (Yeasen) and cOmplete, EDTA-free protease inhibitor cocktail (Roche). The concentration of total protein was determined using the bicinchoninic acid (BCA) kit (Beyotime Biotechnology) followed by standard western blotting procedures.

#### Chemicals

Azacytidine (CAS No. 320-67-2) and decitabine (CAS No. 2353-33-5) were purchased from MedChemExpress. 5-Ad-IAA (CAS No. 2244426-40-0) was a gift from Dr. Xiangbing Qi’s lab at NIBS, Beijing. All of these chemicals were prepared as 10 mM stocks in DMSO (CAS No. 67-68-5) purchased from Sigma-Aldrich and further diluted in DMSO to the desirable concentrations. Polybrene and PEI were purchased from Yeasen. Puromycin and blasticidin were purchased from InvivoGene. Hygromycin B was purchased from Sigma.

#### qPCR

Total RNA was extracted from cells using TRNzol (Tiangen). One microgram of total RNA was reverse transcribed into cDNA using Hiscript III 1st strand cDNA synthesis kit (Vazyme, R312-02) followed by qPCR using *Taq* Pro Universal SYBR qPCR Master Mix (Vazyme, Q712-02). The following primers were used: *FAM126B*-F (5′-TCCCCTCCTTATCCAAGCCT-3′), *FAM126B*-R (5′-ATGCTGACACAATGCCCCTT-3′), *FAM126A*-F (5′-CACGAGTCGAGGTCCTGC-3′), *FAM126A*-R (5′-TCCTCCACAACCCCTTTCTC-3′), *ACTB*-F (5′-CATGTACGTTGCTATCCAGGC-3′), and *ACTB*-R (5′-CTCCTTAATGTCACGCACGAT-3′).

#### Plasmid and cell line construction

The following sgRNAs were cloned into Lenti-guide-puro (Addgene #52963) or Lenti-guide-mNeonGreen-zsGreen (modified from Lenti-guide-puro) using the BsmBI restriction sites: sgChr2-4 (5′-GCAGTGCTAACCTTGCATTG-3′), sgChr2-2 (5′-GGTGTGCGTATGAAGCAGTG-3′), sg*FAM126B* (5′-ACCATTCTTCCACAACACAA-3′), sg*FAM126B*-2 (5′-ACCATTCTTCCACAACACAA-3′), sg*FAM126A* (5′-ATCTCTCTATAAAGTTATCC-3′), sgNTC (5′-GAACTCGTTAGGCCGTGAAG-3′), and sg*POLD3* (5′-GGTTCCGTGACAGACACTGT-3′). P4M sequence was cloned from *Legionella pneumophila* (a gift from Dr. Feng Shao’s lab at NIBS, Beijing). *Sac1* sequence was cloned from *Saccharomyces cerevisiae* (a gift from Dr. Hui Jiang’s lab at NIBS, Beijing). *FAM126B* cDNA was cloned from RKO, mutagenized by introducing synonymous mutations into the sgRNA recognition sites (FAM126B∗) and fused with 3×AID. *OsTIR1*-F74A sequence was a gift from Dr. Lilin Du’s lab at NIBS, Beijing. Lyn11 (5′-ATGGGATGTATAAAATCAAAAGGGAAAGACAGC-3′) and MYR (5′-ATGGGGTCTTCAAAATCTAAACCAAAGGACCCCAGCCAGCGCCGGCGCAGGATCCGAGGTTACCTT-3′) sequences were synthesized as primers. PI4KIIIα cDNA was cloned from RKO. Sequences encoding mCherry-2xP4M, Lyn11-Sac1, FAM126B∗-3×AID, OsTIR1-F74A, Lyn11-BFP were cloned into a lentiviral vector with EF1α core promoter by Gibson assembly. MYR-mCherry, mCherry-PI4KIIIα, and MYR-mCherry-PI4KIIIα were cloned into a piggyBac vector with a CAG promoter by Gibson assembly. Cell lines expressing Cas9, FAM126B∗, FAM126A-3×V5, FAM126B∗-3×AID, and TIR1-F74A were generated with lentiviral infection. Cell lines expressing MYR-mCherry, mCherry-PI4KIIIα or MYR-mCherry-PI4KIIIα were generated using piggyBac transposition.

#### Competitive cell growth and cell viability assays

Cell lines expressing Cas9 were infected with lentivirus expressing sgRNA-mNeonGreen-zsGreen. Three days later, infected cell and uninfected cell were mixed at a ratio of 1:2. Percentages of GFP positive cells were measured by cytometry every three or four days as described.^53^ Direct measurement of cell viability was performed using CellTiter-Glo luminescent cell viability assay kit (Promega, G7571). Luminescence was recorded by EnVison multimode plate reader (PerkinElmer, Waltham, USA).

#### Cell line-derived xenograft

For *in vivo* tumor challenge experiments, 4×10^6^ CRC cells in 125 μL Dulbecco’s phosphate-buffered saline (DPBS, Gibco) were inoculated to 8–10-week-old female BALB/c-Nu mice. Tumor length (L) and width (W) were determined by Vernier caliper at the indicated times, and tumor volumes were calculated by L×W^2^×0.5.

#### CRISPR screening in RKO and DLD1

RKO-Cas9 or DLD1-Cas9 cell lines were infected with lentivirus harboring the human Brunello sgRNA library at low multiplicity of infection (0.2–0.3). Cells were cultured and passaged for 21 days. Genomic DNA was extracted using standard phenol-chloroform extraction. PCR amplification was performed using NEBNext Q5 Hot Start HiFi PCR Master Mix (NEB, M0544L) according to manufacturer’s instructions.^54^ Genes with depleted sgRNAs were analyzed by MAGeCK (Model-based Analysis of Genome-wide CRISPR–Cas9 Knockout).^55^

#### Subcellular fractionation by differential centrifugation

Cells were resuspended with ice-cold hypotonic lysis buffer (20 mM HEPES, 10 mM KCl, 1 mM EDTA, 1 mM EGTA, 2 mM MgCl_2_, 1 mM DTT, supplemented with EDTA-free protease inhibitor cocktail) and incubated on ice for 15 min. Afterward, cell suspension was passed through a 27-gauge needle for 10 times and centrifuged at 800 rcf (4°C) for 10 min. The supernatant was centrifuged at 100,000 rcf (4°C) for 60 min. The resulting supernatant contained the cytosol. The pellet (containing plasma membrane) was dissolved with SDS lysis buffer (20 mM HEPES-NaOH, pH 8.0, 10 mM NaCl, 1 mM EDTA, 2 mM MgCl_2_, and 1% SDS).

#### Detection of PI4P with the mCherry-2×P4M probe

CRC cells expressing Cas9, FAM126B∗-3×AID, OsTIR1-F74A, and Lyn11-BFP were seeded in a cell culture dish with a glass bottom. The mCherry-2×P4M plasmid was transfected into cells with Lipofectamine 3000 (Thermo Fisher Scientific). Cells were treated with 250 nM 5-adamantyl-indole-3-acetic acid (5-Ad-IAA) for 24 h and then imaged with a Nikon SIM confocal microscope. Quantitative analysis of imaging data was performed using ImageJ with the JACoP Plugin.

#### Bioinformatic analysis

DepMap Public 22Q1, including gene effect (Chronos), gene expression (RNA-seq) and cell lines information was downloaded from the DepMap data portal. The list of human gene paralogs was obtained from a previous study.^24^ Chronos scores and expression values for 53 CRC cell lines were extracted. For each gene in the list, a Pearson correlation coefficient and associated *p* value was computed between its Chronos scores versus the expression levels of its paralog. The analysis was performed using R (version 4.1.2) in R Studio (version 2021.09.2 + 382 for Windows). Gene expression data of Cancer Cell Line Encyclopedia (CCLE), TCGA and GTEx were downloaded from UCSC Xena browser. Expression of *FAM126A* and *FAM126B* were grouped into tumor versus normal, or MSI versus MSS according to their sample type annotations. Violin plots were generated by GraphPad Prism (version 8.0) using default parameters. Promoter DNA methylation (methylation fraction 1 kb upstream of transcription start sites) data in CRC cell lines were downloaded from DepMap. Methylation 450k data for TCGA colon adenocarcinoma (COAD) and rectal adenocarcinoma (READ) were downloaded from UCSC Xena browser. *FAM126A* promoter region was defined according to Mexpress.^56^ Sample entries with “NA” were excluded from analysis. Heatmap generation and Pearson correlation analysis were performed using GraphPad Prism (version 8.0). FAM126A and FAM126B protein expression data of The Clinical Proteomic Tumor Analysis Consortium (CPTAC) were downloaded from LinkedOmicsKB (https://kb.linkedomics.org/).^57^^,^^58^ Quantification results of FAM126A and FAM126B protein expression in human colon, rectum and colorectal cancer samples based on immunohistochemistry were downloaded from the Human Protein Atlas (https://www.proteinatlas.org/). Antibodies used in the analysis was HPA042873 and HPA036167.

### Quantification and statistical analysis

Details of sample sizes and statistical tests can be found in the figure legends. All data centers are depicted as mean; dispersion and precision measures can be found in the figure legends. T-test was performed with Prism (version 8.0) or excel (2021 Professional Plus). All correlation analyses were performed with Prism (version 8.0) using the default parameters.
