# Supplementary material for: Frequent loss of FAM126A expression in colorectal cancer results in selective FAM126B dependency
Source: iScience. 2024 Mar 29;27(5):109646. doi: 10.1016/j.isci.2024.109646 (PMC11025007; doi:10.1016/j.isci.2024.109646)
Supplement: Document S1. Figures S1–S9 [file mmc1.pdf]

iScience, Volume *27*

Supplemental information

Frequent loss of *FAM126A* expression in colorectal cancer results in selective *FAM126B* dependency

Shuang Li and Ting Han

Supplementary information

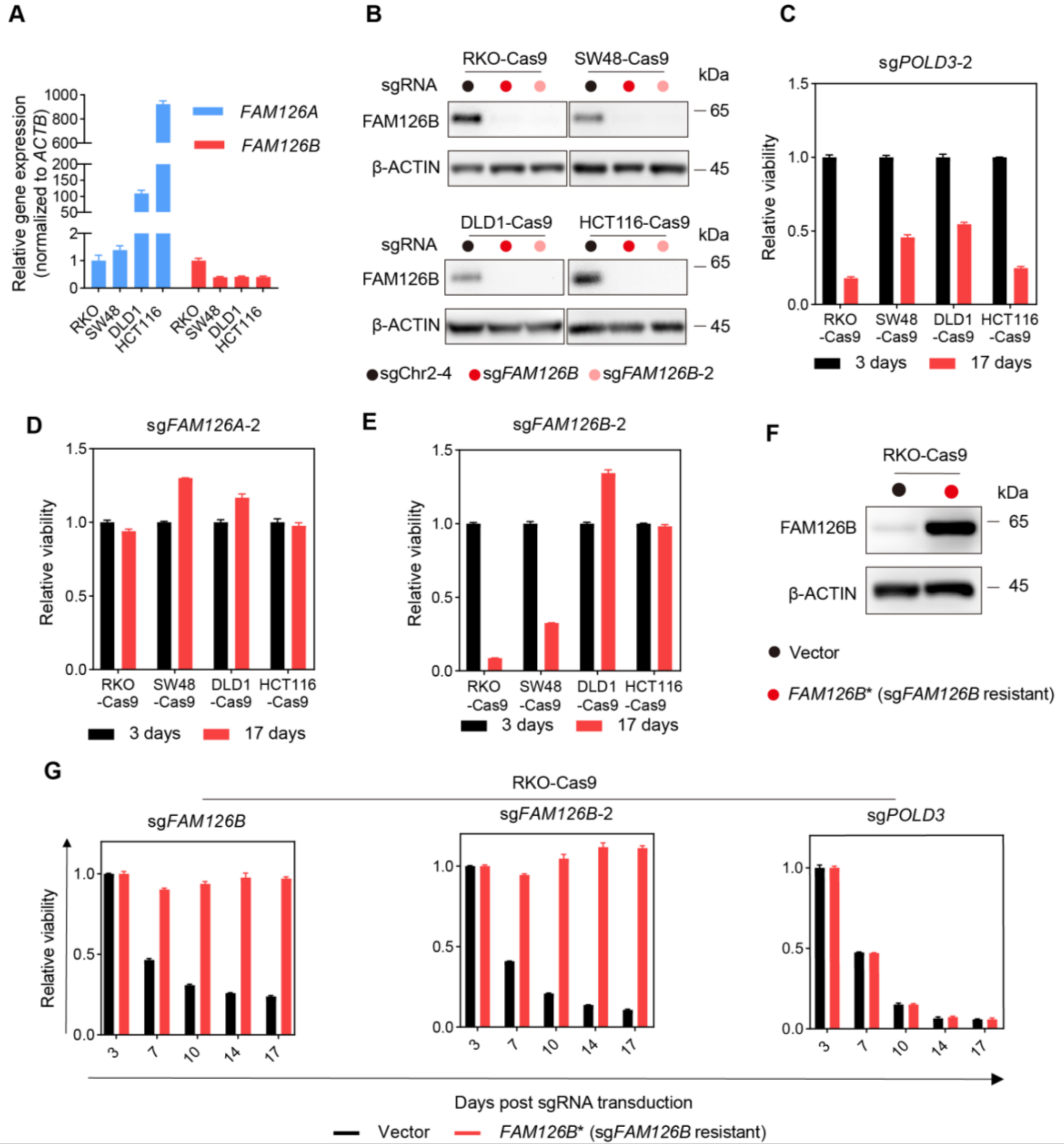

Figure S1. Depletion of FAM126B reduces the viability of FAM126A^{low} CRC cell lines, related to Figure 1. A, qPCR quantification of *FAM126A* and *FAM126B* mRNA levels in indicated cell lines. Data are the mean ± s.d. of three independent samples normalized to *ACTB*. **B**, Depletion of FAM126B by two independent sgRNAs shown by western blotting. **C**. Competitive cell growth assay after inactivation of *POLD3* with an independent sgRNA (sg*POLD3*-2) in indicated cell lines. **D,** Competitive cell growth assay after inactivation of *FAM126A* with an independent sgRNA (sg*FAM126A*-2) in indicated cell lines. **E**, Competitive cell growth assay after inactivation of *FAM126B* with an independent sgRNA (sg*FAM126B*-2) in indicated cell lines. **F**, Detection of FAM126B in RKO-Cas9 cells expressing vector or an sgRNA-resistant mutant of *FAM126B* (*FAM126B**) by western blotting. **G**, Competitive cell growth assay after inactivation of *FAM126B* in RKO-Cas9 cells expressing vector or *FAM126B**. Data in **C**, **D**, **E**, and **G** are the mean ± s.d. from three technical replicates and normalized to control (sgChr2-4).

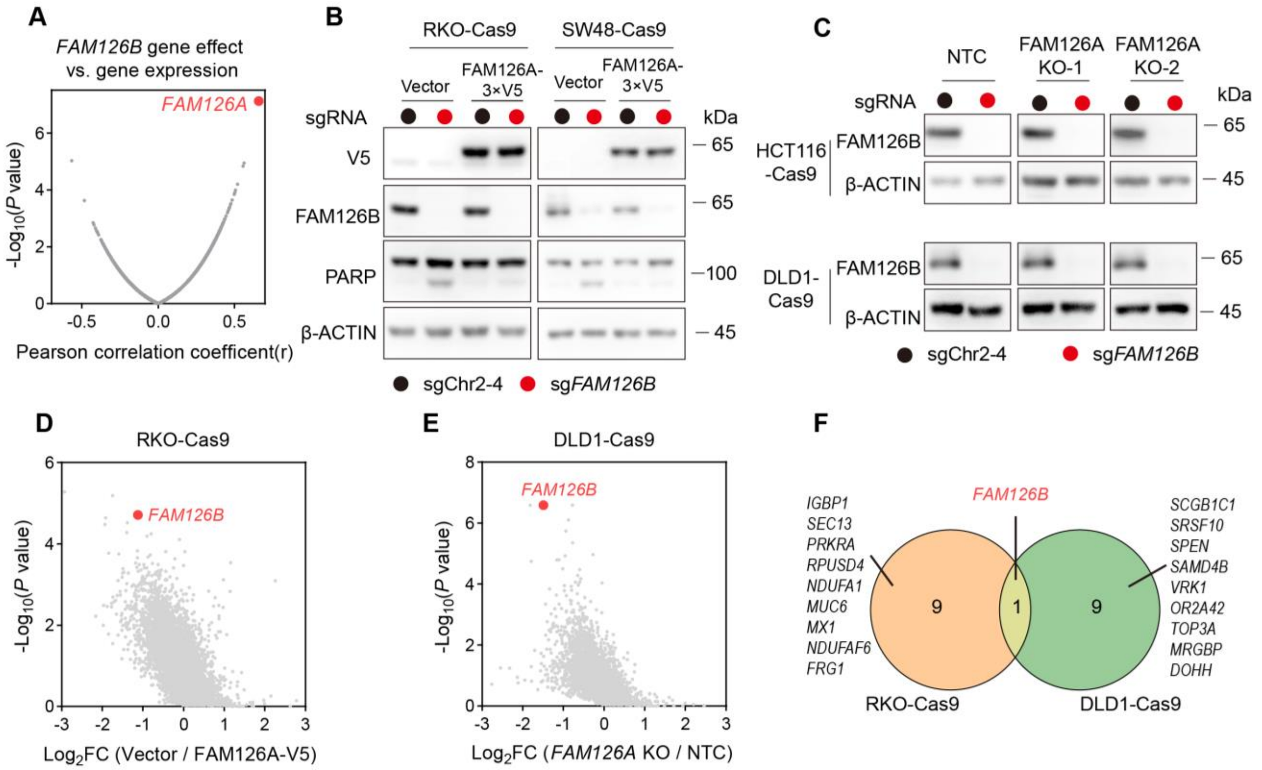

Figure S2. Perturbation of *FAM126A* expression alters FAM126B dependency in CRC cell lines, related to Figure 3. A, Correlation of *FAM126B* gene effect (Chronos) with the expression levels of every human gene among 53 CRC cell lines. **B**, Detection of FAM126B depletion and PARP1 cleavage in indicated cell lines following sgRNA transduction by western blotting. **C**, Verification of FAM126B depletion in indicated cell lines. **D**, Scatterplot depicting genes with significantly depleted sgRNAs in RKO-Cas9 cells expressing vector versus RKO-Cas9 cells expressing FAM126A-3×V5. **E**, Scatterplot depicting genes with significantly depleted sgRNAs in DLD1 *FAM126A* knockout cells relative to DLD1 cells expressing a non-targeting control (NTC) sgRNA. Genes were plotted based on mean $\log_2$ fold change ($\log_2$FC) of sgRNA counts and *P* values computed by MAGeCK. **F**, Venn diagram indicating the overlap between top 10 depleted genes (based on *P* values) in **D** and **E**.

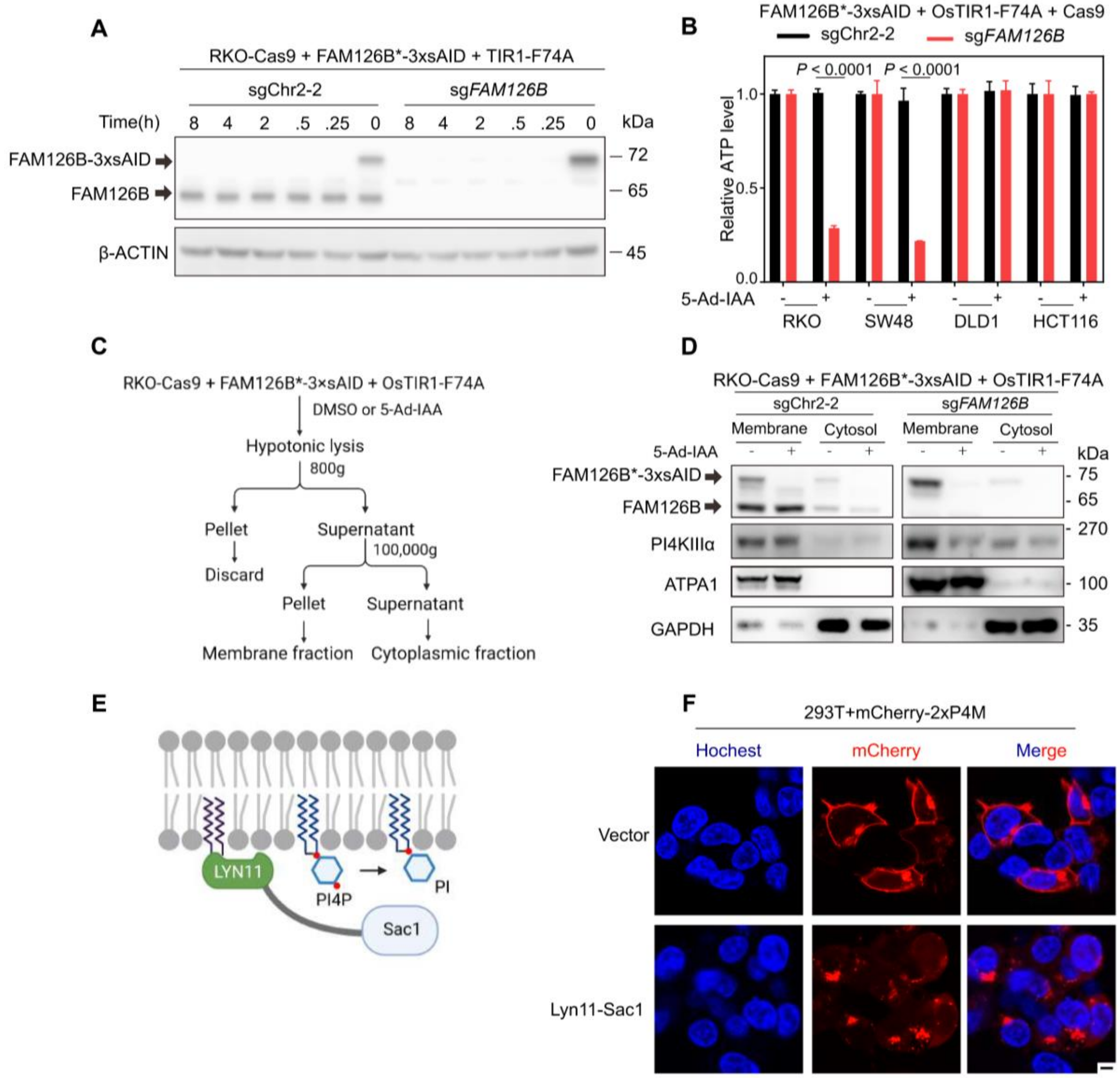

Figure S3. FAM126B is required for PI4KIIIα plasma membrane localization, related to Figure 4. A, Degradation of FAM126B-3×AID induced by 5-Ad-IAA at indicated time points. **B**, Effect of FAM126B-3×AID degradation on cell viability. Indicated cell lines were cultured in the presence of DMSO or 250 nM 5-Ad-IAA for about 10 days before measurement of intracellular ATP. Data are the mean $\pm$ s.d. of three biological replicates and normalized to DMSO. **C**, Flowchart of subcellular fractionation. **D**, Detection of indicated proteins in subcellular fractions as indicated by western blotting. Cells were treated with DMSO or 5-Ad-IAA for 24 hours before subcellular fractionation. ATPA1 (ATPase Na+/K+ transporting subunit alpha 1) was used as a plasma membrane marker. GAPDH (glyceraldehyde-3-phosphate dehydrogenase) was used as a cytosolic marker. **E**, Strategy for depleting PI4P on plasma membrane. **F**, Effect of Lyn11-Sac1 on cellular pools of PI4P. Vector or Lyn11-Sac1 was co-transfected with mCherry-2×P4M into 293T cells. Confocal images were taken 24 hours later. Hochest staining was used to visualize nuclei. Scale bar: 5 μm.

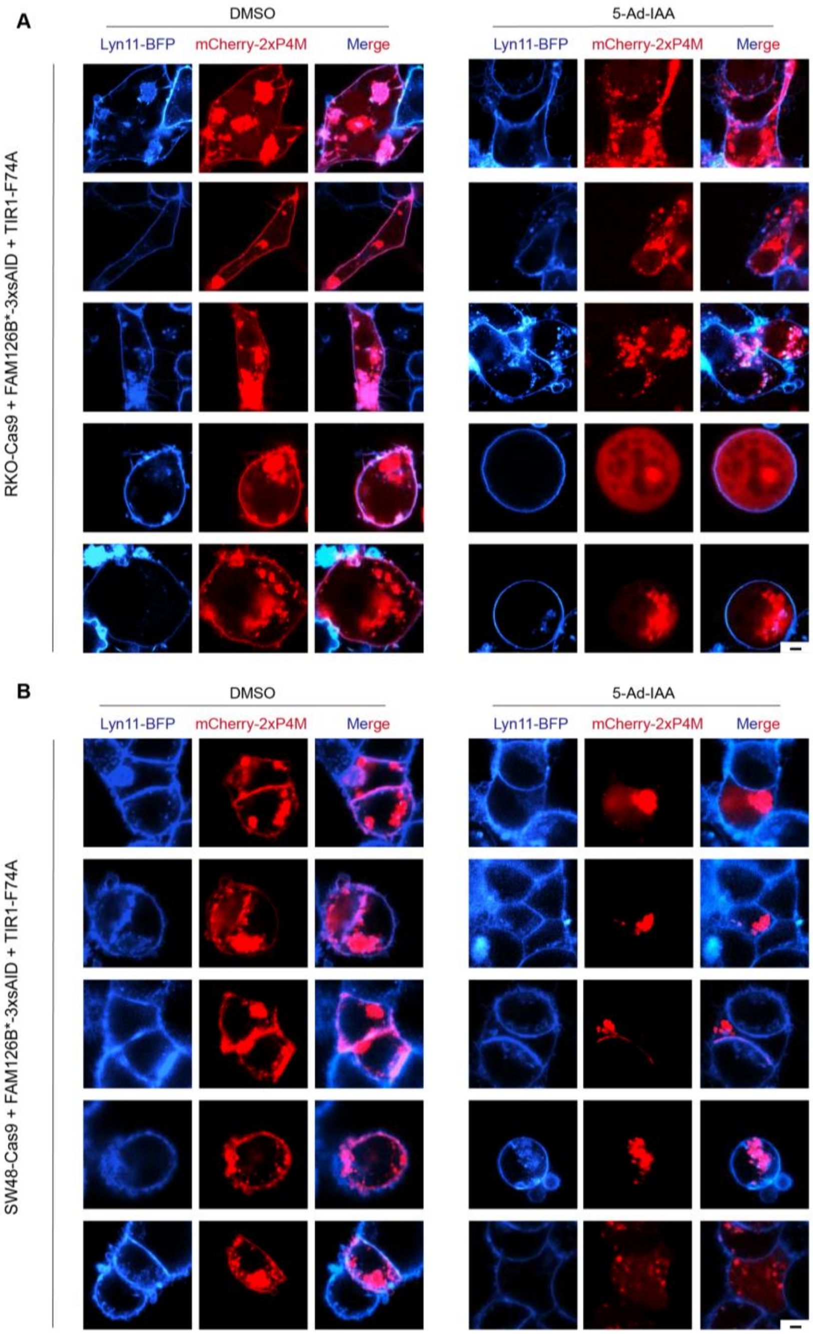

Figure S4. FAM126B degradation depletes the PM PI4P pool in RKO and SW48 cells, related to Figure 4. A–B, Detection of cellular PI4P by transiently transfecting mCherry-2×P4M probe into indicated cell lines followed by confocal imaging. Lyn11-BFP is a plasma membrane marker. Scale bar: 2.5 μm.

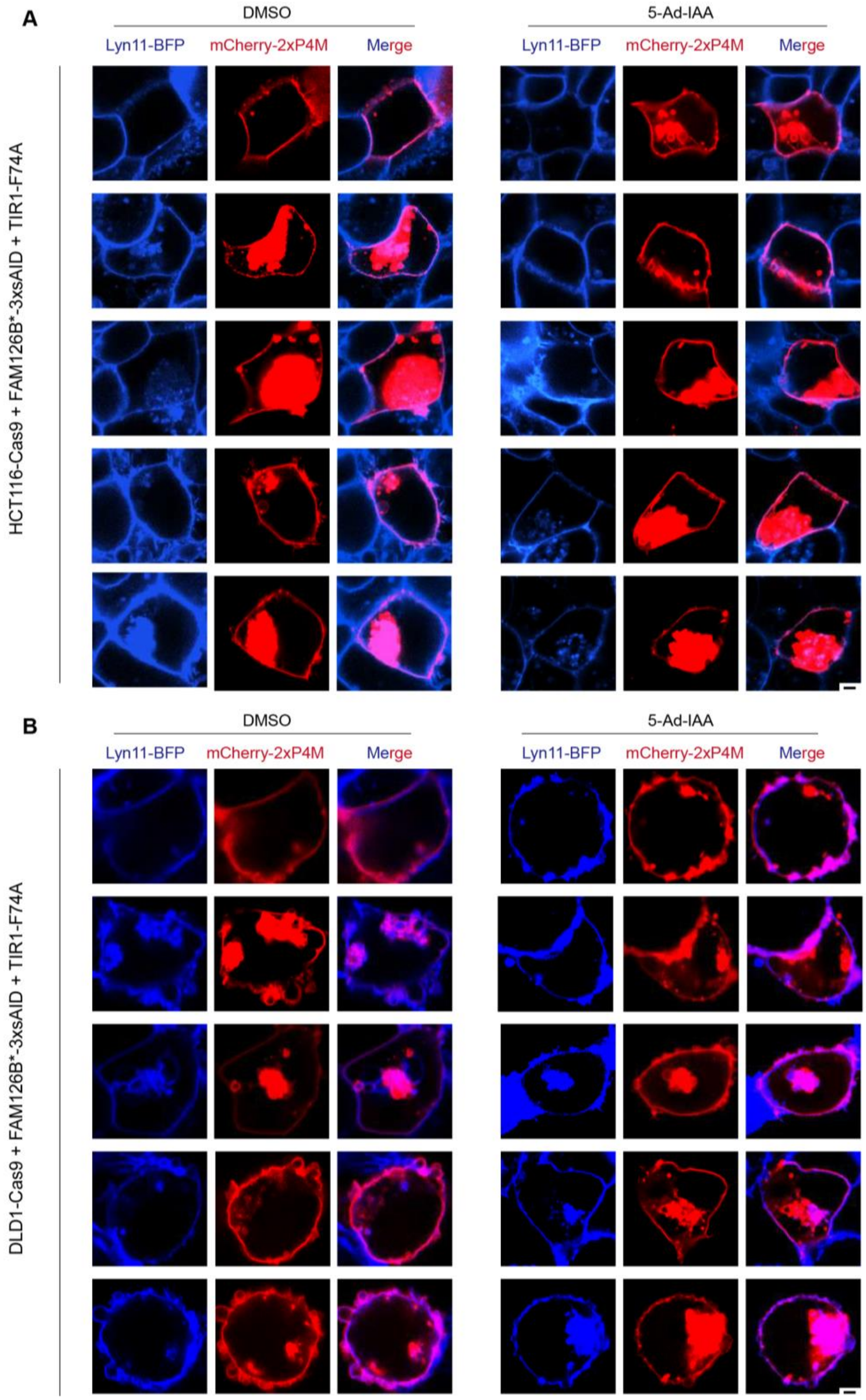

Figure S5. FAM126B degradation does not affect the PM PI4P pool in DLD1 and HCT116 cells, related to Figure 4. A–B, Detection of cellular PI4P by transiently transfecting mCherry-2×P4M probe into indicated cell lines followed by confocal imaging. Lyn11-BFP is a plasma membrane marker. Scale bar: 2.5 μm.

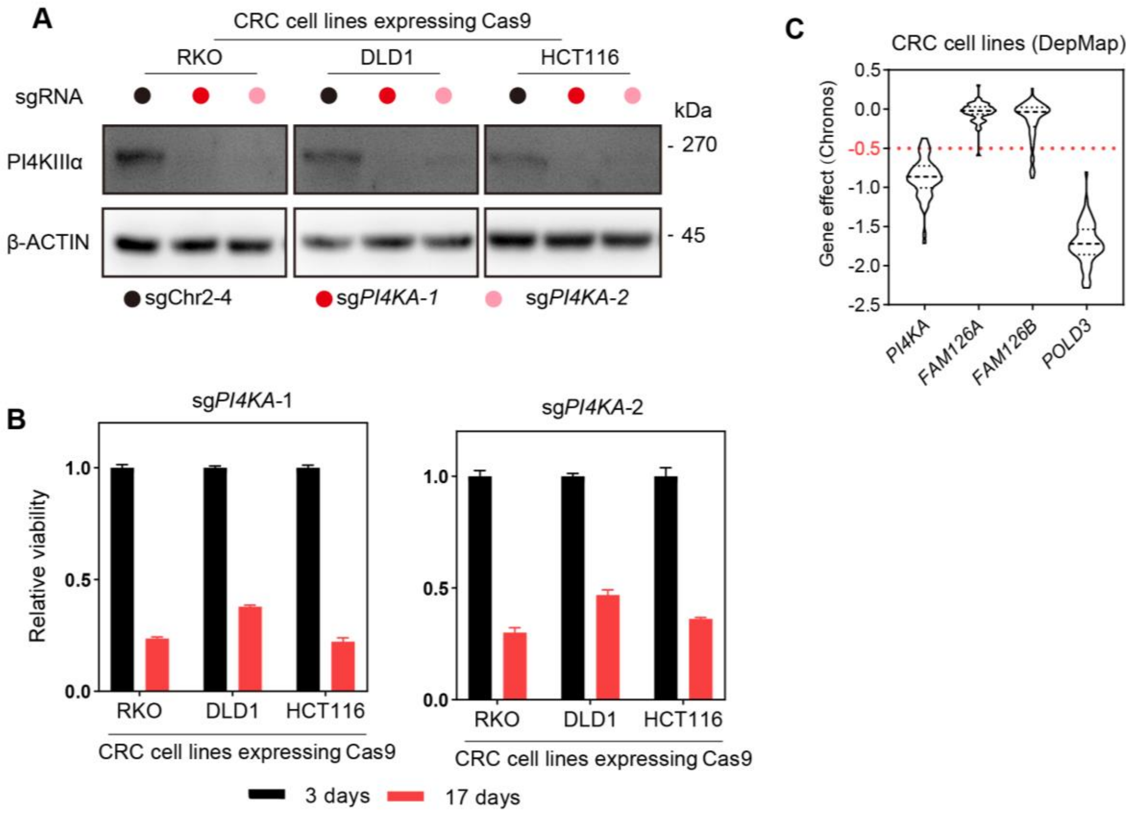

Figure S6. *PI4KA* is a common essential gene, related to Figure 5. A, Verification of PI4KIIIα depletion with two independent sgRNAs by western blotting. **B**, Competitive cell growth assay after inactivation of *PI4KA* in indicated cell lines. Data are the mean ± s.d. from three technical replicates and normalized to control (sgChr2-4). **C**, Violin plot depicting the distribution of the gene effects of *PI4KA*, *FAM126A*, *FAM126B*, and *POLD3*.

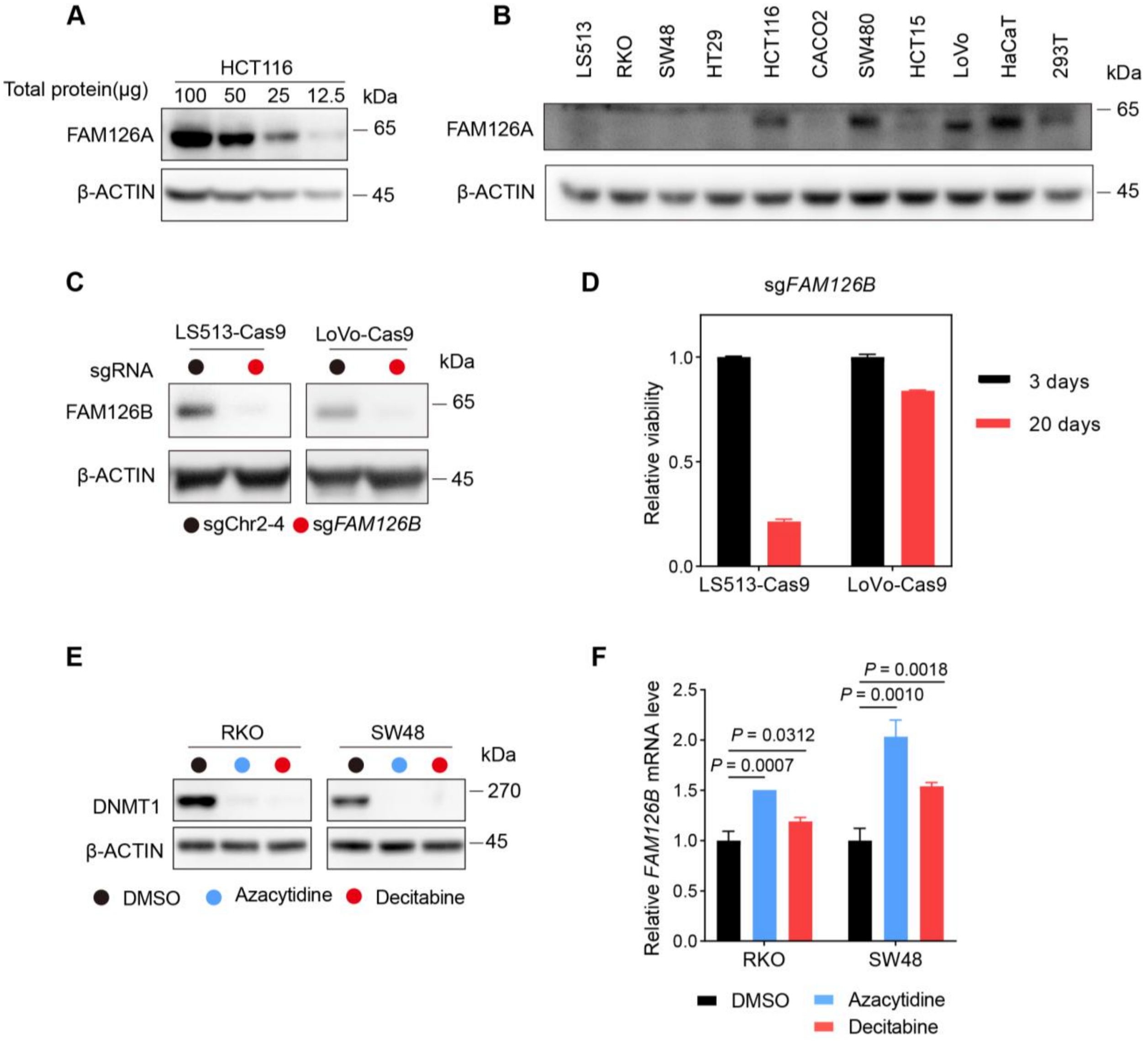

Figure S7. Epigenetic silencing of *FAM126A* expression is prevalent and can be reversed by inhibition of DNA methylation in CRC cell lines, related to Figure 6. A, Measurement of the linear range of FAM126A detection by western blotting. **B**, Detection of FAM126A in indicated cell lines by western blotting. **C**, Verification of FAM126B depletion in indicated cell lines. **D**, Competitive cell growth assay after inactivation of *FAM126B* in indicated cell lines. Data are the mean ± s.d. from three technical replicates and normalized to control (sgChr2-4). **E**, Detection of DNMT1 following treatment with azacytidine or decitabine of indicated cell lines by western blotting. **F**, Effect of azacytidine and decitabine on *FAM126B* expression. RKO or SW48 cells were treated with 4 μM Azacytidine or 20 μM decitabine for 72 hours before qPCR analysis of *FAM126A* expression. Student's t-tests (two-tailed, unpaired) were used to determine the statistical significance. Data were the mean ± s.d. of three biological replicates.

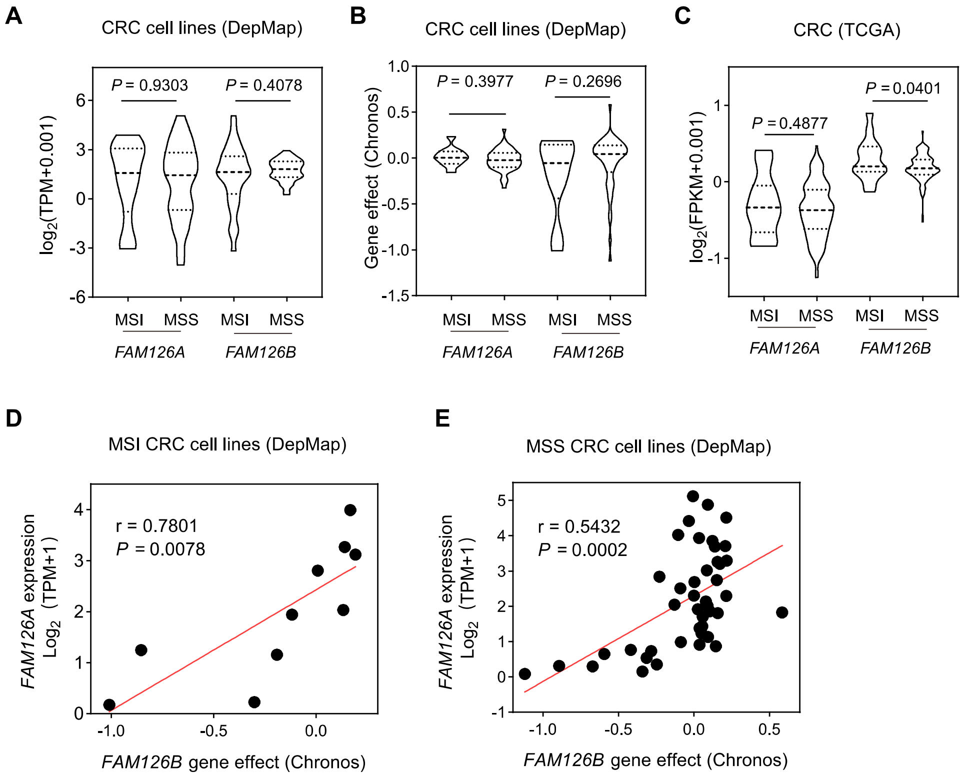

Figure S8. SL relationship between *FAM126A* and *FAM126B* in MSI and MSS CRC cell lines, related to Figure 6. A, Violin plot depicting the distribution of *FAM126A* and *FAM16B* expression in MSI (n=16) and MSS (n=56) CRC cell lines from DepMap (22Q4). **B**, Violin plot depicting the distribution of *FAM126A* and *FAM16B* dependence in MSI (n=10) and MSS (n=46) CRC cell lines from DepMap (22Q4). **C**, Violin plot depicting distribution of *FAM126A* and *FAM126B* expression in MSI (n=15) and MSS(n=114) CRC tumor samples. Data were obtained from TCGA. **D**, Scatterplot depicting the correlation between *FAM126A* expression and *FAM126B* gene effect in MSI CRC cell lines (n=10). Pearson correlation coefficient (r) and *P* value were indicated on the plot. Linear regression was represented by the red line. **E**, Scatterplot depicting the correlation between *FAM126A* expression and *FAM126B* gene effect in MSS CRC cell lines (n=43). Pearson correlation coefficients (r) and *P* values were indicated on the plot. Linear regression was represented by the red line.

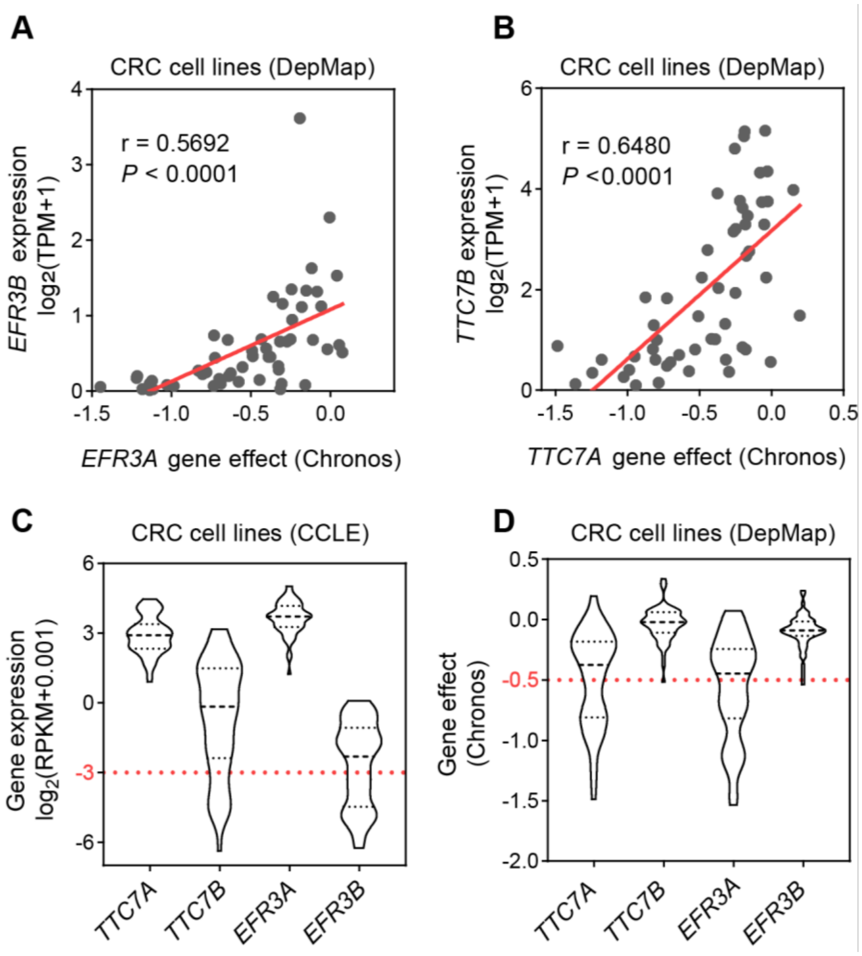

Figure S9. SL interactions in *EFR3* and *TTC7* gene families, related to Figure 6. A, Scatterplot depicting the correlation between *EFR3B* expression and *EFR3A* gene effect. **B**, Scatterplot depicting the correlation between *TTC7B* expression and *TTC7A* gene effect. TPM stands for transcripts per million clean reads. Pearson correlation coefficient (r) and *P* value were indicated on the plot. Linear regression was represented by the red line. **C**, Violin plot depicting the distribution of *EFR3A/B* and *TTC7A/B* expression in 57 CRC cell lines from CCLE. **D**, Violin plot depicting the distribution of *EFR3A/B* and *TTC7A/B* gene effects (Chronos) in 57 CRC cell lines from DepMap (22Q4).
